# Efficacy and Safety of the Biosimilar Recombinant Human Parathyroid Hormone Cinnopar^®^ in Postmenopausal Osteoporotic Women: A Randomized Double-blind Clinical Trial

**Published:** 2018-09

**Authors:** Ozra TABATABAEI-MALAZY, Masumeh NORANI, Ramin HESHMAT, Mostafa QORBANI, Afsaneh VOSOOGH, Behnaz AFRASHTEH, Farzin KAHKESHAN, Arman AJAMI, Bagher LARIJANI

**Affiliations:** 1. Non-Communicable Diseases Research Center, Endocrinology and Metabolism Population Sciences Institute, Tehran University of Medical Sciences, Tehran, Iran; 2. Endocrinology and Metabolism Research Center, Endocrinology and Metabolism Clinical Sciences Institute, Tehran University of Medical Sciences, Tehran, Iran; 3. Osteoporosis Research Center, Endocrinology and Metabolism Clinical Sciences Institute, Tehran University of Medical Sciences, Tehran, Iran; 4. Chronic Diseases Research Center, Endocrinology and Metabolism Population Sciences Institute, Tehran University of Medical Sciences, Tehran, Iran; 5. Non-communicable Diseases Research Center, Alborz University of Medical Sciences, Karaj, Iran; 6. Faculty of Pharmacy, Tehran University of Medical Sciences, Tehran, Iran

**Keywords:** Teriparatide, CinnoPar®, Postmenopausal osteoporosis, Clinical trial

## Abstract

**Background:**

Due to high cost and burden of osteoporosis, it is reasonable to focus on the reduction of fractures as the main goal of treatment. We compared the efficacy and safety of a new biosimilar recombinant human parathyroid hormone (CinnoPar®, CinnaGen, Iran) to the reference product (Forteo®, Eli Lilly, USA) in a randomized double-blind clinical trial (RCT).

**Methods:**

Overall, 104 osteoporotic postmenopausal women aged 45–75 yr were randomized to receive 20 μg daily subcutaneous injections of either Forteo® or CinnoPar® for 6-months from 2011–2012. Bone biomarkers were measured at baseline, and during first, third, and sixth month’s follow-up along with lumbar spine, total hip, and femoral neck bone mineral density (BMD) assessment at the baseline and six months after that. The study was registered in Iranian registry of clinical trials under the registration number of IRCT138810121414N5. The endpoints were to compare bone biomarkers, BMD and drug safety between groups. Data analysis was performed using SPSS 11.

**Results:**

Age range of ninety-four patients who completed the study was 42–81 yr. Participants were divided into Forteo (45 subjects) and CinnoPar (49 subjects) groups. No significant difference in terms of bone biomarkers or BMD scores was shown between groups (*P*≥0.05). The most prevalent side effects were hypercalcemia and hypercalciuria without any significant statistical differences between groups.

**Conclusion:**

CinnoPar® can be considered as a good alternative therapy for Forteo® in postmenopausal osteoporotic women due to its comparable efficacy and safety properties.

## Introduction

Osteoporosis, as a major metabolic bone disorder, is characterized by reduced bone density and the deterioration of skeletal microarchitecture (1). The prevalence of osteoporosis is increasing both in developed and developing countries (2–4). According to WHO and using dual-energy x-ray absorptiometry (DEXA), osteoporosis is defined by a bone mineral density (BMD) that is 2.5 standard deviations below the average value of the mean peak young adults bone mass (5).

Recently, International Osteoporosis Foundation (IOF) has estimated the number of 200 million osteoporotic women in the world where a fragility fracture is estimated to occur every 3 sec (2). Prevalence of osteoporosis is influenced by a range of different risk factors (6). It is prevalent among women (34% versus 17% in men), especially in postmenopausal women (2). About two million people in Iran are at risk of fracture (3). Osteoporosis can significantly lead to bone fragility, disability, and fracture risk (7). Thus, its diagnosis and appropriate treatment is the main challenge worldwide (8, 9). With respect to accelerating rate of burden and costs attributable to osteoporosis, a deeper focus to set fracture reduction as the main treatment goal is highlighted. Most of the currently approved agents for the prevention and treatment of osteoporosis are anti-resorptive and anabolic agents (4, 10–12).

Since BMD changes often should be measured at least one year following treatment with rhPTH, bone biochemical markers can provide valuable clinical information and early feedback for monitoring of treatment (12). Short-term changes in Procollagen Type 1 N-Terminal Propeptide (P1NP), as a bone formation biomarker, are a good alternative for BMD in postmenopausal osteoporotic women who received teriparatide for a year (13). Similarly, the early markers of bone formation can be suggestive of BMD and bone architecture changes following 2 yr of teriparatide treatment in osteoporotic postmenopausal women (14, 15). Bone Marker Standards Working Group has recently suggested measuring C-terminal cross-linking telopeptide (CTX) along with P1NP in clinical studies, as specific markers of bone resorption and formation, respectively (13).

Teriparatide (Forteo®), as the recombinant human parathyroid hormone (rhPTH), [1–34], has been approved by Food and Drug Administration (FDA) for the treatment of fracture-prone post-menopausal osteoporotic women and men. Teriparatide administrated subcutaneously once-daily exerts its effect by preferentially inducing bone formation over bone resorption resulting in a net accumulation of bone mass (11). However, due to the high cost and restrictive policies, many patients have limited access to this therapy. These concerns along with the patent expiration of Forteo^®^ in Jul 2013 encouraged the development of biosimilars for the reference product. CinnoPar® manufactured by CinnaGen pharmaceutical company (Karaj, Iran), is introduced as a teriparatide biosimilar. Biosimilars are expected to demonstrate similar quality to the reference product in terms of comparable functional and structural properties.

Therefore, this trial aimed to compare the efficacy and safety of teriparatide biosimilar (CinnoPar®) to that of reference product (Forteo®) among postmenopausal osteoporotic women.

## Materials and Methods

This randomized, double-blind, and parallel study was reported based on Consolidated Standards of Reporting Trials (CONSORT) 2010. Participants were postmenopausal osteoporotic women attending in the outpatient clinic of Endocrinology and Metabolism Research Center (EMRC) of Dr. Shariati Hospital (Tehran, Iran). The period of study was from Feb 2011 through Oct 2012.

Inclusion criteria were postmenopausal women aged 45–75 yr who had T-score <−3 by BMD measurement through DEXA scan in at least one of the areas of lumbar spine, femoral neck or total hip without any fracture or T-score <−2.5 in at least one of the three aforementioned areas and past history of osteoporotic fracture after a minor trauma in the vertebrae or extremities. Exclusion criteria were lack of consent to participate in the trial and not complying with treatment and follow-up procedures, past receiving of bone resorption inhibitors for at least four weeks, corticosteroid-usage ≥6 months before recruitment, hypercalcemia (above 10 mg/dl) or hypercalciuria with calcium to creatinine ratio ≥1, history of receiving PTH and Strontium, history of recurrent nephrolithiasis, autoimmune, advanced liver or kidney disorders.

This study was approved by the ethics committee of EMRC as the institutional review board (IRB) with a reference code of 87/11/161. All the procedures were in compliance with the principles of Good Clinical Practice (GCP) and the declaration of Helsinki. The study was registered in Iranian registry of clinical trials under the registration number of IRCT138810121414N5.

Sample size calculation for 80% power, α= 0.05, sample size ratio =1 and expected mean difference= −0.05 by following equation (n= (Z_α/2_+Z_β_)^2*^2*δ^2^/d^2^) was 52 in each group.

Informed consent was obtained from all 104 eligible patients. Then patients were randomized to receive 20 mg daily subcutaneous injections of either biosimilar recombinant human PTH (CinnoPar®, CinnaGen, Iran) or the innovative product (Forteo®, Eli Lilly, USA) by permuted balanced block randomization using Excel software (blocks of four, allocation ratio 1:1) for six months. Randomization was performed by an independent party not involved elsewhere in the trial. Concealment of allocation was performed by sequentially numbered, sealed, opaque, and stapled envelopes. Separate persons were responsible for generation of randomization codes, and treatment allocation. Both products were relabeled and recorded in order to become indiscernible by the appearance. All the participants in this study, including patients, physicians, and nurses were blinded to the treatment allocation.

According to the evidence-based recommendations to PTH therapy (16), all of patients took daily 1000 mg of elemental calcium one month before rhPTH initiation until the end of study plus 300000 IU vitamin D at baseline that followed by 400 IU daily during the study. The primary and secondary endpoints were to compare bone biomarkers along with total hip, femoral neck, lumbar spine BMD, and drug safety between groups.

Efficacy was evaluated at baseline, first, third and sixth months follow-up by bone biomarkers including P1NP, Bone-Specific Alkaline Phosphatase (BSAP), Osteocalcin (OC), C-terminal telopeptide of type I collagen (CTX), and N-terminal telopeptide of type I collagen (NTX) along with serum calcium, PTH and 25-hydroxyvitamin D (25-OHD). Patients’ urine samples were taken from their second-morning urine.

Laboratory tests’ assessments were ELISA for bone biomarkers and 25-OHD levels (IDS, UK), ELISA for PTH (Biomerica, USA), and autoanalyzer for serum calcium (Parsazmoon, Iran) that performed in a single center. BMD was performed through DEXA scan in a single center.

All statistical analyses were based on the intention-to-treat sample and performed using SPSS software (ver. 11, Chicago, IL, USA). The normal distribution of data was evaluated by Kolmogorov-Smirnov analytic test. Data were analyzed by Chi-Square, General Linear Models (multivariate), and Paired T-Test. *P*≤0.05 was considered statistically significant.

## Results

**Fig. 1:**
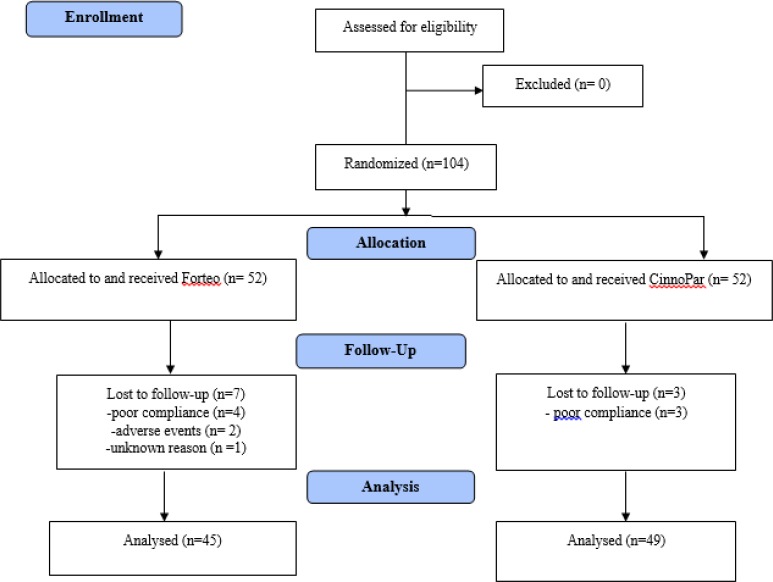
Trial flow diagram based on CONSORT 2010

From 104 patients, seven patients dropped out of the study in the Forteo® arm for reasons, including poor compliance (n=4), adverse events (n=2), and unknown reason (n=1); whereas in the CinnoPar® arm dropouts were three patients due to poor compliance. Trial flow diagram is shown based on CONSORT 2010 in Fig. 1. Finally, the Forteo and CinnoPar groups included 45 and 49 osteoporotic women, respectively. Participants aged 42–81 yr with a mean ± SD of 62.82 ±8.62. The baseline characteristics are summarized in Table 1.

**Fig. 2:**
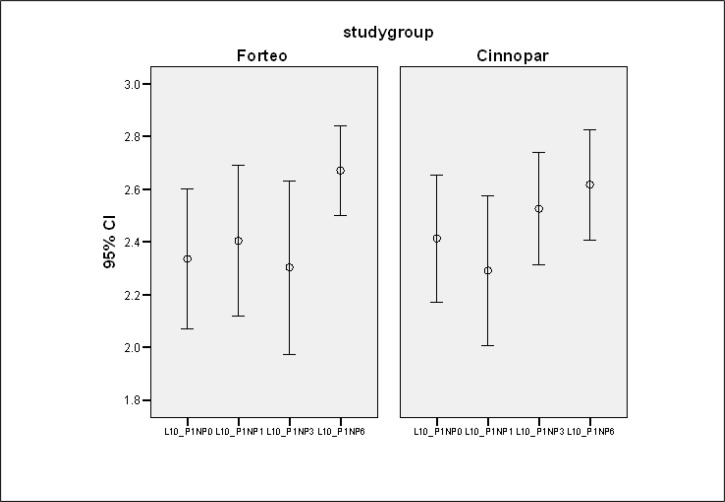
CI95% of P1NP changes over the time

Patients had comparable baseline characteristics regarding P1NP, P1CP, BSAP, OC, CTX, and NTX, along with lumbar spine, femoral neck, and total hip BMD that presented as mean ± SD in Table 2 and Fig. 2.

**Table 1:** Baseline characteristics of the patients in the Forteo® and CinnoPar® groups

***Variable***		***CinnoPar^®^ (n=49)***	***Forteo^®^ (n=45)***	***P***
Educational level; n (%)	Literate	25 (51.0)	21 (46.7)	0.87
	Primary education	16 (32.7)	17 (37.8)	
	University education	8 (16.3)	7 (15. 6)	
History of fracture in mother; n (%)	Yes	10 (20.4)	13 (29.5)	0.38
	No	38 (77.6)	30 (68.2)	
Family history of osteoporosis; n (%)	Yes	20 (41. 7)	21 (47.7)	0.54
	No	28 (58.3)	23 (52.3)	
Cigarette smoking; n (%)	Current smoker	0 (0.0)	1 (2.2)	0.53
	Previous smoker	3 (6.5)	4 (8.9)	
	Never	43 (93.5)	40 (88. 9)	
Alcohol consumption; n (%)	Previous alcohol consumption	0 (0.0)	2 (4.4)	0.24
	Never	47 (100.0)	43 (95. 6)	
Drug abuse; n (%)	Previous abuse	2 (4.3)	3 (6.7)	0.48
	Never	45 (95.7)	42 (93.3)	

Statistical analysis was Chi-square and *P*≤0.05 was considered as statistically significant.

**Table 2:** Baseline and time tend of bone biomarkers and biochemical tests in treated groups

***Variable***	***Treatment***	***Measurement***				***Trend***	***Time interaction***
		**Baseline**	**First month**	**3^rd^ month**	**6^th^ month**		
P1NP (pg/ml)	a	218.78±5.25	251.19±5.75	199.53±7.59	467.74±2.82	0.035	0.43
	b	257.04±4.90	194.98±6.46	338.84±4.07	416.87±3.98		
BSAP (unit/lit)	a	26.92±1.58	32.36±1.48	37.15±1.38	44.67±1.48	<0.001	0.58
	b	30.90±1.58	36.31±1.58	44.67±1.62	56.23±1.91		
CTX (pg/ml)	a	275.42±1.42	309.03±1.55	363.08±1.45	407.38±1.38	<0.001	0.93
	b	251.19±1.82	275.42±1.82	338.84±1.58	371.54±1.55		
OC (ng/ml)	a	3.72±1.99	5.25±2.51	5.50±2.88	5.13±2.69	<0.001	0.55
	b	3.16±1.99	4.90±2.57	5.62±2.88	3.98±1.95		
NTX (nmol)	a	15.85±1.41	17.78±1.48	22.91±1.48	24.55±1.51	<0.001	0.28
	b	16.60±1.48	19.95±1.51	28.84±1.58	27.54±1.78		
C1CP (nmg/ml)	a	53.70±1.99	70.79±1.86	70.79±1.86	69.18±2.24	<0.001	0.19
	b	64.57±1.82	107.15±1.99	107.15±1.99	91.20±2.24		
Serum Ca (mg/dl)	a	9.33±1.05	9.77±1.05	9.77±1.05	9.77±1.07	<0.001	0.68
	b	9.33±1.05	9.55±1.05	9.77±1.07	9.77±1.07		
Urine Ca (mg/dl)	a	190.55±1.48	186.21±1.95	190.55±1.86	141.25±1.74	0.002	0.17
	b	158.49±1.66	169.82±2.24	251.19±1.51	141.25±1.74		
25-OHD (nmol/L)	a	77.62±2.51	89.13±2.24	81.28±2.09	81.28±1.86	0.14	0.69
	b	70.79±2.19	85.11±1.74	81.28±1.66	83.18±1.74		
PTH (pg/ml)	a	38.02±1.91	22.91±1.95	20.89±1.78	21.38±1.86	<0.001	0.41
	b	41.69±1.86	29.51±1.95	23.99±1.99	21.88±2.04		

Legend: a: Forteo, b: CinnoPar, P1NP: Procollagen Type 1 N-Terminal Propeptide, BSAP: Bone-Specific Alkaline Phosphatase, CTX: C-terminal cross-linking telopeptide, OC: Osteocalcin, NTX: N-terminal telopeptide of type 1 collagen, C1CP: C-Propeptide of Type 1 Procollagen, Ca: Calcium, 25-OHD: 25 hydroxyvitamin D, NS: non-significant

Statistical analysis was General Linear Models (multivariate), and *P*≤0.05 was considered as statistically significant

Data are presented as mean± SD

Although, there was a significant difference in the mean percentage changes in bone biomarkers concentration within groups, changes in over the time were not statistically significant between groups (*P*>0.05).

Mean percentage changes in BMD measures of total hip, lumbar spine, and femoral neck after six months of treatment is shown non-statistically significant differences between groups, Table 3.

**Table 3:** Baseline and time trend of changes in BMD in treated groups

***BMD***	***Treatment***	***Mean ±SD***		***Mean differences (%) ± SE***	***P***	***Power (%)***
		**Baseline**	**End of study**			
Total hip	a	1.82±0.68	1.80±0.63	0.9 ± 1.9	NS	40
	b	1.82±0.76	1.72±0.78	6.3 ± 2.4		
Lumbar-Spine	a	3.32±0.73	3.04±0.73	8.3 ± 1.8	NS	5.3
	b	3.14±0.77	2.85±0.75	8.7 ± 1.3		
Femoral Neck	a	2.24±0.70	2.19±0.72	1.7 ± 2	NS	5.5
	b	2.12±0.66	2.10±0.69	2.3 ± 2		

Legend: a: Forteo, b: CinnoPar, NS: non-significant.

Statistical analysis was Paired *t*-test, *P*≤0.05 was considered as statistically significant

The incidence of hypercalcemia and hypercalciuria (>300 mg per day), as important safety concerns of rhPTH therapy was not statistically significant between groups (*P*=0.91 and *P*=0.92, respectively). Other reported adverse effects were bone pain, hypotension, tachycardia, vomiting, and headache.

## Discussion

The results of this RCT demonstrated non-statistical significant differences in the efficacy of CinnoPar^®^ and Forteo^®^ on bone biomarkers as well as BMD. Also, CinnoPar^®^ has comparable efficacy and safety profile to Forteo^®^ in post-menopausal osteoporotic women.

In a seminal study, around 1600 postmenopausal women with previous vertebral fractures were exposed to daily teriparatide (20 μg or 40 μg) or placebo for 21 months. By both doses of teriparatide, the total body bone mineral (2%–4%) improved and vertebral and non-vertebral fractures were reduced (60%) compared to placebo (14). Overall, evidence have shown an increasing trend in the BMD of spine and femoral neck by 8.6%–13%, and 3.5%–6%, respectively, as well as a reduction of 65%–69% and 53% in the risk of vertebral and non-vertebral fractures, respectively (12, 16–18). In the meanwhile, no immediate reduction in fracture risk followed by the initiation of rhPTH therapy was reported due to the fact that the induced changes in bone structure and increasing bone density and strength are expected in long-term. In a placebo-trial study conducted to assess fracture risk in non-vertebral bones, fracture risk reduction was failed to occur until 9 to 12 months after initiation of teriparatide (12). Therefore, in the present study, bone biomarkers were assessed to investigate the effect of teriparatide. Several studies have evaluated the clinical responses to short-term teriparatide intake. Almost all of these studies have used lumbar spine BMD and bone biomarkers as measures for outcome efficacy (19–23). Similarly, we assessed treatment response to rhPTH with these parameters, and non-significant differences were observed in lumbar spine, femoral neck, and total hip BMD between treated groups. Similar to ours, another study has suggested an increase in the lumbar BMD even after one-month rhPTH therapy (24). However, a long-term follow-up is recommended for assessing the efficacy of a product on BMD to avoid a possible error in interpreting patient outcomes. In this study, the increments for the mean percentage change in the lumbar spine BMD is in agreement with those obtained in previous trials on postmenopausal osteoporotic women (20, 23, 25). The values for mean change in femoral neck and total hip are also consistent with other studies (22).

Bone biomarkers could be considered as a good indicator of the drug effect on rate of future fractures. In some evidence, a positive correlation was found between initial increase in the concentration of bone biomarkers and the subsequent response of bone density increment after daily injection of PTH (13, 26). Since the beneficial effects of PTH on bone biomarkers are presented in the first month of intake (15), bone biomarkers could be used instead of BMD to evaluate the treatment outcomes in the near future (6-3 months). While this time for BMD is 18-12 months (27). In previous studies, the beneficial effects of PTH on bone resorption or bone formation biomarkers were reported in all of the studied patients (28, 29). However, due to length of drug intake, the beneficial effects could be both include decreasing to increasing of the bone biomarkers (28).

We evaluated multiple bone formation (P1NP, BSAP) and resorption markers (CTX, NTX) to compare the effects of biosimilar and reference product on bone metabolism. In our study, a significant trend in the concentrations of the studied bone biomarkers was marked. The calculated values for the mean percentage change in P1NP, as an important bone formation marker, or in CTX, as a bone resorption marker, were in line with those of previous studies (30–32). However, the important point is to determine the level of changes as significant or non-significant differences between groups. In our study, we observed non-statistically significant difference in any of the aforementioned bone biochemical markers between two products.

Usually, rhPTH is well tolerated in most patients and its side effects are often mild without any treatment requirement. Moreover, the two major short-term side effects are hypercalcemia and hypercalciuria (16). Transient mild hypercalcemia reported in 1%–3% of patients could be mediated by decreasing the intake of calcium or vitamin D (12, 33). In our study, calcium level in plasma and its excretion in urine were exerted. We did not notice any significant differences in the incidence of these parameters between groups. Just in one of our patients, an improvement of hypercalcemia and hypercalciuria after ceasing consumption of calcium and vitamin D was reported, and intermittent injection was used instead of daily injection of rhPTH. The beneficial effects of intermittent injection or daily dose of 10 μg were shown in several studies (23, 34, 35). Other reported adverse effects included transient orthostatic hypotension, tachycardia, nausea, headache, dizziness, leg cramps, and hyperuricemia (36). Similar to our results, no any statistically significant differences were shown for the above adverse effects between under treatment groups from placebo (16). The most important adverse effect of long-term rhPTH is osteogenic sarcoma. A number of experimental studies have reported osteogenic sarcoma followed by treatment with teriparatide in a dose and duration dependent pattern; however, such a trend was not found in patients consuming very trivial dosages of teriparatide (37). Of the 300000 postmenopausal women consumed PTH worldwide, osteogenic sarcoma was reported only in one case (38). Since our study was conducted in a short time period (6 months), only short-term adverse effects of rhPTH therapy were evaluated.

Nevertheless, this study had some limitations. First, BMD should often be evaluated at least one year after treatment intervention. In fact, the 6-month period is quite short to investigate the changes in BMD along with fracture risk reduction. In addition, the sample size in this study was relatively small, and we did not take race parameter into account as an influential factor.

## Conclusion

The low-cost and availability of biosimilar products can improve patients’ access to high-quality treatment options. In addition, daily (20 μg) subcutaneous injections of biosimilar (CinnoPar®) and reference rhPTH product (Forteo®) have comparable efficacy and safety profiles in post-menopausal osteoporotic women. In order to further evaluate long-term effectiveness and side effects of biosimilar rhPTH product, we recommend post-marketing studies to be conducted as a complementary phase of the study.

## Ethical considerations

Ethical issues (Including plagiarism, informed consent, misconduct, data fabrication and/or falsification, double publication and/or submission, redundancy, etc.) have been completely observed by the authors.
